# Antiviral Lipopeptide-Cell Membrane Interaction Is Influenced by PEG Linker Length

**DOI:** 10.3390/molecules22071190

**Published:** 2017-07-15

**Authors:** Marcelo T. Augusto, Axel Hollmann, Matteo Porotto, Anne Moscona, Nuno C. Santos

**Affiliations:** 1 Instituto de Medicina Molecular, Faculdade de Medicina, Universidade de Lisboa, Av. Prof. Egas Moniz, 1649-028 Lisboa, Portugal; maugusto@fm.ul.pt (M.T.A.); ahollmann@medicina.ulisboa.pt (A.H.); 2 Laboratory of Molecular Microbiology, Institute of Basic and Applied Microbiology, National University of Quilmes, Roque Sáenz Peña N° 352, Bernal, 1876 Buenos Aires, Argentina; 3 Laboratory of Biointerfaces and Biomimetic Systems, CITSE, National University of Santiago del Estero-CONICET, 4200 Santiago del Estero, Argentina; 4 Center for Host-Pathogen Interaction, Columbia University Medical Center, 701 W. 168th, New York, NY 10032, USA; mp3509@cumc.columbia.edu; 5 Department of Pediatrics, Columbia University Medical Center, 701 W. 168th, New York, NY 10032, USA; 6 Department of Microbiology & Immunology, Columbia University Medical Center, 701 W. 168th, New York, NY 10032, USA; 7 Department of Physiology & Cellular Biophysics, Columbia University Medical Center, 701 W. 168th, New York, NY 10032, USA

**Keywords:** paramyxoviruses, peptides, antiviral, cholesterol, membranes

## Abstract

A set of lipopeptides was recently reported for their broad-spectrum antiviral activity against viruses belonging to the *Paramyxoviridae* family, including human parainfluenza virus type 3 and Nipah virus. Among them, the peptide with a 24-unit PEG linker connecting it to a cholesterol moiety (VG-PEG24-Chol) was found to be the best membrane fusion inhibitory peptide. Here, we evaluated the interaction of the same set of peptides with biomembrane model systems and isolated human peripheral blood mononuclear cells (PBMC). VG-PEG24-Chol showed the highest insertion rate and it was among the peptides that induced a larger change on the surface pressure of cholesterol rich membranes. This peptide also displayed a high affinity towards PBMC membranes. These data provide new information about the dynamics of peptide-membrane interactions of a specific group of antiviral peptides, known for their potential as multipotent paramyxovirus antivirals.

## 1. Introduction

Human respiratory diseases caused by the *Paramyxoviridae* family of viruses are a serious worldwide concern that affect the human population in all of its strata. Children under age 5, elderly adults, and immune-compromised individuals are high risk groups, due to their insufficient antiviral defenses. Human parainfluenza viruses (HPIVs), either respiroviruses (HPIV1 and HPIV3) or rubulaviruses (HPIV2 and HPIV4), are common causes of significant lower respiratory tract disease including pneumonia. Documented HPIV infection accounts for 30–40% of all acute respiratory tract infections in children [1]. The zoonotic paramyxovirus Nipah virus (NiV) emerged in the human population from its bat reservoir via pig intermediate hosts, but has now been transmitted directly between humans and represents a global health risk [2]. Human infection results in a range of outcomes from asymptomatic infection to a severe acute respiratory disease and fatal encephalitis, with neurological sequelae years later. There is no available treatment to prevent or treat human parainfluenza infection. For henipaviruses, monoclonal antibodies have been developed for use in human infection, but are not feasible for widespread use in the field under the conditions of an outbreak [3].

Traditionally, antiviral drugs have been designed to target viral proteins or host cell factors that are involved in the infection process, including the characterization of the involved viral and cellular targets. The first step of viral infection, however, requires the involvement of a conserved structure, the lipid membrane. Two families of photoactivatable molecules were recently found to biophysically modify the viral membrane and render the virions inert due to the absence of an internal membrane repair system [4,5,6,7,8]. Both families have a broad spectrum of activity and could theoretically target any enveloped virus. In the future, it may be possible to improve the efficacy of such compounds in vivo, minimize their toxicity, assess possible neutralizing effects by the cell membrane, and establish whether these compounds can be photoactivated inside the human body [9].

Some membrane-active peptides have been reported to disrupt the membranes of bacteria and fungi as part of their antimicrobial mechanism of action [10]. A subgroup of these molecules, virolytic antiviral peptides, can preferentially target the lipid membranes of a wide range of viruses while sparing cell membranes [11,12]. The selectivity of these peptides for distinct membrane compositions is a topic of ongoing investigation. 

Our previous studies revealed the importance of the specific lipid bilayer membrane to the mechanism of action of antiviral peptides [13,14,15,16,17], as also reviewed elsewhere [18]. Peptides designed to target the viral entry glycoprotein of a specific virus were found to be active against related viruses, resulting in broad-spectrum activity [19,20].

Recently, we developed these antiviral peptides as a promising strategy for prevention and treatment of infection by HPIV or NiV [20,21]. Entry of virus into the host cell during the initial steps of infection by both viruses is initiated by recognition of a receptor in the host cell membrane by a viral surface glycoprotein and activation of the viral fusion protein (F), which extends via a conformational rearrangement to insert its hydrophobic fusion peptide into the host cell membrane. At this point, viral and target cell membranes are bridged by a transient F intermediate that refolds into an energetically stable structure, an antiparallel six-helix bundle, thought to be responsible for driving fusion of the viral and cell membranes. The transient intermediate structure of F can be targeted by fusion inhibitory peptides either at the N-terminal repeat (HRN) or at the C-terminal repeat (HRC), corresponding to the two domains that must interact to form the six-helix bundle. Peptides derived from the HRC domain of HPIV3 F were found to be very effective at inhibiting fusion and viral entry mediated by HPIV3 and also by NiV [21]. We recently showed that conjugation of cholesterol and PEG24 to a fusion inhibitory peptide derived from this HPIV3-HRC peptide (referred to as “VG” to distinguish it from other versions of this peptide) resulted in broad-spectrum antiviral activity [21]. This strategy has been proved to be useful not only for HPIV and NiV but also for other enveloped viruses including HIV, measles virus and influenza viruses [21,22,23,24,25,26,27,28].

In this study, we evaluated the interaction of C-terminally lipid conjugated VG peptides [21] with membranes of different composition and with human peripheral blood mononuclear cells (PBMC). Our data indicate that the lipid-conjugated peptides interact with cholesterol-rich membranes, likely to be important domains for viral infection. Moreover, the high peptide-membrane affinity obtained for PBMC indicates that these peptides can circulate within the host attached to cells, potentially increasing their half-life and efficacy.

## 2. Results and Discussion

Recently, we showed that peptides derived from the HRC domain of paramyxovirus F proteins have broad-spectrum antiviral activity, inhibiting fusion and entry mediated by HPIV3 and NiV [21]. Conjugating the peptides with cholesterol (Chol) and linkers made of polyethyleneglycol (PEG) led to better inhibition and the development of a potent lipopeptide, VG-PEG24-Chol. Here we investigated whether a set of VG peptides (Table 1) remain membrane active when challenged to interact with membranes rich in cholesterol, an important membrane component that is present in lipid raft micro-domains [29,30]. Membranes composed of cholesterol and also sphingolipids form these highly ordered rigid domains with limited fluidity in comparison to the surrounding plasma membrane. Lipid rafts are thought to be involved in fusion and budding of different enveloped viruses namely HIV-1 [31], influenza [32,33] and paramyxovirus [34,35,36]. Respiratory viruses that invade the airway epithelium activate a humoral and cellular immune response with inflammatory cell recruitment [37,38,39]. Leukocytes serve as a spreading vehicle for Nipah virus, which can efficiently bind to PBMC and travel within the host to infect new tissues [40]. We thus addressed the interaction of VG peptides with PBMC as a model for what may happen in the circulation.

### 2.1. PEG Linker Influences Peptide-Lipid Interactions

**Figure 1:**
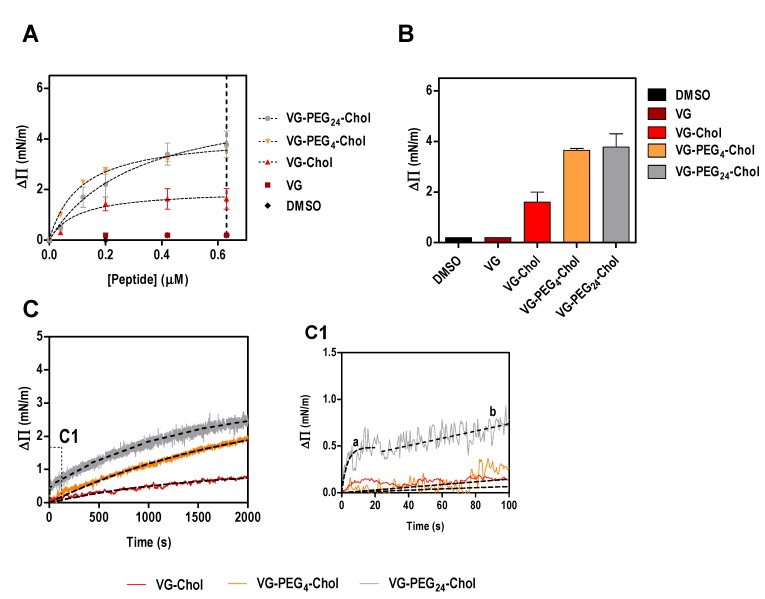
Surface pressure perturbation of lipid monolayers. (**A**) Changes in the surface pressure as a function of concentration of VG peptides (or DMSO) added to POPC:Chol 2:1 monolayers. (**B**) Maximum surface pressure perturbation achieved at 0.63 µM of peptide (dashed line in A) or DMSO. (**C**) Variation of the surface pressure of POPC:Chol 2:1 monolayers as a function of time after injection of VG peptides at a final concentration of 0.2 µM. **C1** is a section of the first 100 s of the kinetic assay showing the two-phase behavior of VG-PEG24-Chol, characterized by a fast membrane insertion during the first 20 s (**a**), followed by a slower interaction over time (**b**).

Using a simplistic model of lipid monolayers composed solely of POPC, we have shown that the membrane affinity of VG peptides is similar among the cholesterol-conjugated peptides [21]. The absence of cholesterol in this model membrane might explain why no significant differences were observed in peptide affinities. To address this possibility, in this work we evaluated the ability of VG peptides to induce changes on the surface pressure of lipid monolayers composed of POPC and cholesterol in a 2:1 ratio. As expected, VG peptide did not induce a change in the lipid monolayer surface pressure (Figure 1). On the other hand, all the cholesterol-conjugated peptides induced changes in the surface pressure of lipid monolayers (Figure 1), until a plateau is reached, likely due to saturation with the peptide or limited peptide-membrane effect. This result reinforces the importance of the lipid moiety for the interaction. The fitting of the experimental data using Equation (1), enabled the calculation of the apparent dissociation constant, K_d_. Interestingly, the peptide that showed least affinity to the membrane was VG-PEG24-Chol (Table 2), despite inducing the largest change in surface pressure along with VG-PEG4-Chol (Figure 1B). The maximum surface pressure change induced by the peptides (ΔΠ_max_) seems not to be affected by the linker length. However, the kinetic insertion rate for VG peptides (*k*), calculated using Equation (2), still describes fast kinetics of interaction for VG-PEG24-Chol, as previously observed for pure POPC membranes [21]. In fact, the dynamic interaction that PEG24 confers on the peptide was confirmed in this experiment, where a moderate membrane affinity that does not restrict the fast kinetics of peptide attachment and detachment from the lipid membrane is optimal. For VG-PEG24-Chol, a two-phase kinetics is observed (Figure 1C, section C1), in which a highly fast membrane insertion occurs for the first 20 s, followed by a moderate interaction for the rest of the assay. The perturbation of the lipid bilayer by PEG [41], together with the increased membrane absorption of longer polymers [42], may explain why VG-PEG24-Chol shows a better insertion rate than VG-PEG4-Chol. 

### 2.2. HPIV3 HRC Peptides Interact with PBMC

**Figure 2:**
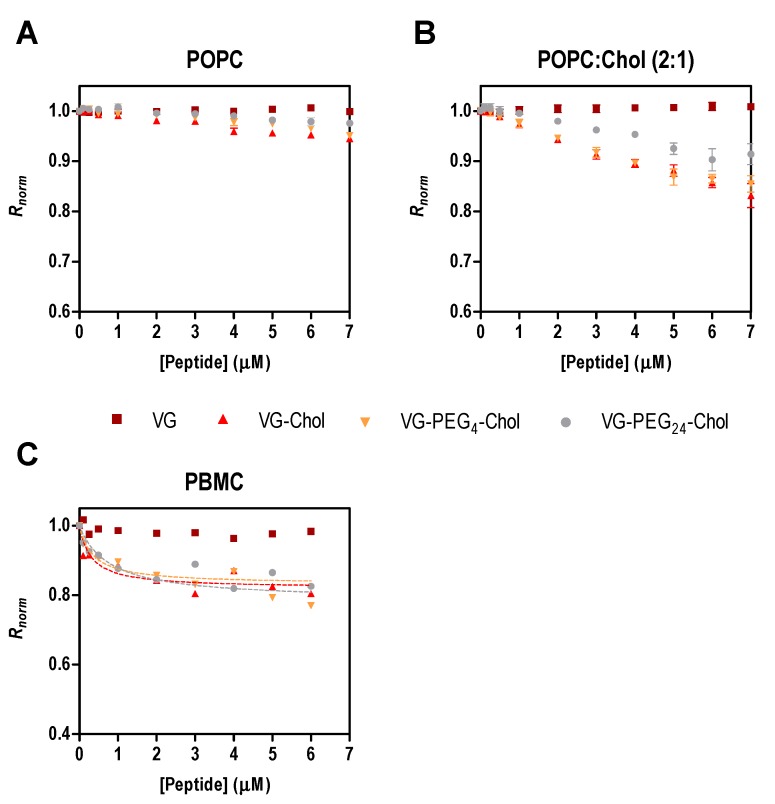
Fusion inhibitory peptides’ interaction with di-8-ANEPPS labeled liposomes and cells. Binding profiles of VG peptides to LUVs of POPC (**A**) or POPC:Chol 2:1 (**B**), and to human PBMC (**C**), obtained by plotting the di-8-ANEPPS excitation ratio *R* (I_455_/I_525_, normalized to the initial value), as a function of peptide concentration.

To assess the affinity of the peptides for cell membranes, we chose PBMC due to their potential role in viral dissemination, as mentioned above. As the direct measurement of peptide tryptophan fluorescence is impracticable with cells, an indirect reporter sensitive to membrane dipole potential was used [15,21,24,26]. For the sake of comparison with surface pressure data, the initial experiments were assessed with di-8-ANEPPS labelled liposomes of POPC and POPC:Chol 2:1 in the presence of different concentrations of the peptides. In Figure 2A,B, the perturbation of the membrane dipole potential in the presence of all cholesterol-tagged peptides was higher in membranes with cholesterol than in those with pure POPC. In agreement with the pressure data (Table 2 and [21]), the highest changes in surface pressure (ΔΠ_max_) were obtained in cholesterol-containing membranes. These data indicate that membrane composition influences the peptides’ behavior in the lipid bilayers. Recently, we showed that two membrane fusion inhibitory peptides of measles virus perturb membranes composed of POPC:Chol 2:1 more than those composed of pure POPC [28], a trend that was also apparent for HIV fusion inhibitory peptides [26].

PBMC were isolated from human blood samples, labeled with di-8-ANEPPS and incubated for 1 h with a range of peptide concentrations. Only the cholesterol-conjugated peptides induced changes in the membrane dipole potential sensed by the fluorescent probe (Figure 2C). The unconjugated VG peptide and the DMSO control (data not shown) did not cause variations in the measured parameter, as found for the liposome assays. To quantify the interaction between the VG peptides and PBMC, the ratio *R* of the fluorescence intensities with emission at 670 nm and excitation wavelengths at 455 nm and 525 nm was measured and used to calculate the dissociation constant, K_d_, using Equation (3). *R* decreases upon increasing the concentration of cholesterol-tagged peptides, while the unconjugated VG peptide does not induce any change in the membrane dipole potential of PBMC. VG-PEG24-Chol showed a higher affinity (K_d_ = 0.36 µM) in comparison to VG-PEG4-Chol (K_d_ = 0.77 µM), but similar to VG-Chol (K_d_ = 0.32 µM). Despite the small variation in the dissociation constants (Table 3), they are not significantly different, indicating that these cholesterol-conjugated peptides have a similar affinity towards PBMC membranes.

## 3. Materials and Methods

### 3.1. Peptide Synthesis and Lipids

All peptides were produced by standard Fmoc-solid phase methods. The cholesterol moiety was attached to the peptide via chemoselective reaction between the thiol group of an extra cysteine residue, added C-terminally to the sequence, and a bromoacetyl derivative of cholesterol, as previously described [21,22,43]. POPC (1-palmitoyl-2-oleoyl-*sn*-glycero-3-phosphocholine) was purchased from Avanti Polar Lipids (Alabaster, AL, USA), while cholesterol (Chol) was from Sigma (St. Louis, Missouri, MO, USA).

### 3.2. Surface Pressure

Changes in the surface pressure of lipid monolayers induced by VG, VG-Chol, VG-PEG4-Chol or VG-PEG24-Chol were measured in a NIMA ST900 Langmuir-Blodgett trough (NIMA, Coventry, UK), at constant temperature (25 ± 0.5 °C). Briefly, a solution of lipids in chloroform was spread on a Teflon trough of fixed area until it reached a surface pressure of 23 ± 1 mN/m. Peptide solutions were injected in the subphase and the changes in surface pressure were followed for the time necessary to reach a constant value. The surface pressure of an air-water interface upon injecting the largest concentration of each peptide used throughout the studies was always below 15 mN/m. For this reason, the lowest initial surface pressure of the lipid monolayers before the addition of the peptides to the subphase was above that value. In this condition, the changes in surface pressure observed upon the injection of the peptide can be attributed to an effect of the peptide on the monolayer’s interfacial tension. The apparent dissociation constant (K_d_) was calculated from the adsorption Langmuir isotherm:

(1) $$∆\Pi =\frac{∆\Pi_{max}\left(peptide\right)}{K_{d}+\left(peptide\right)}$$

where ΔΠ is the change of surface pressure, ΔΠ_max_ is the maximum change of surface pressure achieved and [peptide] is the peptide concentration. The insertion rate constant (*k*) was calculated from the ΔΠ vs. time (t) data, using the equation:

(2) $$∆\Pi =-e^{-kt}∆\Pi_{max}+∆\Pi_{max}$$

### 3.3. Membrane Dipole Potential Assessed by Di-8-ANEPPS

Membrane dipole potential studies were based on fluorescence spectroscopy measurements carried out in a Varian Cary Eclipse fluorescence spectrophotometer (Mulgrave, Australia). HEPES and NaCl were from Merck (Darmstadt, Germany). The working buffer used throughout the studies was HEPES 10 mM pH 7.4 in NaCl 150 mM. All fluorescence measurements were performed at approximately 25 °C. Human blood samples were obtained from healthy volunteers, with their previous written informed consent, at Instituto Português do Sangue (Lisbon, Portugal), with the approval of the joint Bioethics Committee of Faculdade de Medicina da Universidade de Lisboa and Hospital de Santa Maria. All methods were performed in accordance with the relevant guidelines and regulations. Isolation of peripheral blood mononuclear cells (PBMC) and labeling of these cells with di-8-ANEPPS (Invitrogen, Carlsbad, CA, USA) were performed as previously described [15,44]. PBMC were isolated by density gradient using Lymphoprep (Axis-Shield, Oslo, Norway) and counted in a MOXI Z Mini Automated Cell Counter (Orflo Technologies, Ketchum, ID, USA). Cells were incubated at 3000 cells/µL in Pluronic-supplemented buffer with 3.3 µM di-8-ANEPPS during 1 h, with gentle agitation. Unbound probe was washed with Pluronic-free buffer on two centrifugation cycles. VG peptide series were incubated with PBMC at 100 cells/µL, during 1 h, with gentle agitation, before the fluorescence measurements. For lipid vesicle labeling, suspensions with 500 µM of total lipid were incubated overnight with di-8-ANEPPS 10 µM, to ensure maximum incorporation of the probe. The maximum concentration of DMSO in the suspensions was 2.4% (*v*/*v*) at 6 µM of peptide or in the controls (cholesterol). Excitation spectra and the ratio of intensities at the excitation wavelengths 455 and 525 nm (*R* = I_455_/I_525_) were obtained with emission set at 670 nm, in order to avoid membrane fluidity-related artifacts [45,46]. Excitation and emission slits for these measurements were set to 5 and 10 nm, respectively. The variation of *R* with the peptide concentration was analyzed by a single binding site model [47]:

(3) $$\frac{R}{R_{0}}=1+\frac{\frac{R_{min}}{R_{0}}\left(peptide\right)}{K_{d}+\left(peptide\right)}$$

With *R* values normalized for *R*_0_, the value in the absence of peptide. *R*_min_ defines the asymptotic minimum value of *R* and K_d_ is the apparent dissociation constant.

### 3.4. Data Analysis and Fitting

All the analysis and data fitting were performed in Prism 5 (GraphPad Software, La Jolla, CA, USA).

## 4. Conclusions

The biophysical data shown here support the conclusions obtained in our previous work, where VG-PEG24-Chol was identified among the VG series of HRC-derived peptides as the optimal inhibitor of HPIV3 and NiV virus infection and also as the molecule with highest affinity towards POPC membranes and inducing the most extensive changes in membrane surface pressure [21]. In the present study, we included cholesterol as a component of membranes to study membranes that are more ordered than those composed of pure POPC, a feature that could modulate the peptides’ biological activity. All the cholesterol-conjugated peptides interact with cholesterol-rich model membranes and also with human PBMC, expanding upon the data obtained previously for this set of peptides. The membrane affinity of VG-PEG24-Chol is less for POPC:Chol 2:1 than for pure POPC, but with a significant increment in the kinetic rate of insertion. Interestingly, the kinetic behavior observed for VG-PEG24-Chol with POPC:Chol membranes shows rapid insertion in the membrane in the first 20 s, followed by a slower membrane binding after that initial time period. This kinetics favors the dynamic interaction suggested in our previous work [21], where a flipping of the peptide between the viral and host cell membranes may explain the antiviral efficacy of VG-PEG24-Chol. The addition of cholesterol to the antiviral peptide is the driving force that anchors the peptides in the membrane, but the PEG24 linker modulates peptide-membrane affinity and promotes the more dynamic interaction of VG-PEG24-Chol, a feature that correlates with antiviral activity.

## Figures and Tables

**Table 1:** HPIV3 HRC derived peptide sequences. The residues highlighted in red were modified from the original HPIV3 F protein.

Peptide Name	Sequence and Modifications (N-to-C)
HPIV3 F protein residues 449–484	VALDPIDISIELNKAKSDLEESKEWIRRSNQKLDSI
VG	Ac-VALDPIDISIVLNKAKSDLEESKEWIRRSNGKLDSI-GSGSG-C-NH_2_
VG-Chol	Ac-VALDPIDISIVLNKAKSDLEESKEWIRRSNGKLDSI-GSGS-C(Chol)-NH_2_
VG-PEG4-Chol	Ac-VALDPIDISIVLNKAKSDLEESKEWIRRSNGKLDSI-GSGS-C(PEG4-Chol)-NH_2_
VG-PEG24-Chol	Ac-VALDPIDISIVLNKAKSDLEESKEWIRRSNGKLDSI-GSGS-C(PEG24-Chol)-NH_2_

**Table 2:** Peptide-lipid monolayer interaction parameters. The data from Figure 1A were fitted using Equation 1, yielding the maximum surface pressure change, ΔΠ_max_, and the dissociation constant, K_d_, values presented here. The data on variation of the surface pressure of POPC:Chol 2:1 monolayer as a function of time after injection of VG peptides at a final concentration of 0.2 µM (Figure 1C) were fitted with Equation 2, permitting the calculation of the kinetic adsorption rate constant, *k*. Values are means ± standard error of the mean (SEM) of at least 3 experiments.

	VG-Chol	VG-PEG4-Chol	VG-PEG24-Chol	
ΔΠ_max_ (mN/m)	1.26 ± 0.38	4.16 ± 0.16	5.01 ± 0.80	
K_d_ (10^−2^ µM)	9.83 ± 1.59	10.90 ± 1.59	30.89 ± 14.52	
*k* (10^−4^ s^−1^)	2.06 ± 0.01	4.96 ± 0.05	2417 ± 767 ^1^	7.34 ± 0.09 ^2^

^1^ The fit range was the first 20 s; ^2^ the fit ranged from 20–2000 s

**Table 3:** Peptide affinity towards PBMC, assessed by di-8-ANEPPS fluorescence. The dissociation constant, K_d_ and the asymptotic minimum value of *R*, *R*_min_, were obtained by fitting the experimental data to Equation (3). Values are means ± SEM of at least 3 experiments.

Peptides	K_d_ (µM)	*R*_min, norm_
VG	-	-
VG-Chol	0.32 ± 0.11	−0.18 ± 0.01
VG-PEG4-Chol	0.77± 0.38	−0.21 ± 0.03
VG-PEG24-Chol	0.36 ± 0.14	−0.17 ± 0.01
